# *NIC1* cloning and gene editing generates low‐nicotine tobacco plants

**DOI:** 10.1111/pbi.13694

**Published:** 2021-09-23

**Authors:** Qiulin Qin, Matt Humphry, Tijs Gilles, Anne Fisher, Barunava Patra, Sanjay Kumar Singh, Dandan Li, Shengming Yang

**Affiliations:** ^1^ Department of Plant & Soil Sciences University of Kentucky Lexington KY USA; ^2^ Global Leaf R&D Plant Biotechnology British American Tobacco Cambridge UK; ^3^ Kentucky Tobacco Research and Development Center University of Kentucky Lexington KY USA; ^4^ Present address: Aardevo B.V. Johannes Postweg 8 8308 PB Nagele Netherlands; ^5^ Present address: AdvanceBreed Plant Breeding & Genetics Consultancy 33 Queensway, Exning Newmarket CB8 7EU UK; ^6^ Present address: Department of Plant Pathology North Dakota State University Fargo ND 58102 USA; ^7^ Present address: USDA‐ARS Cereals Research Unit Edward T. Schafer Agriculture Research Center Fargo ND 58102 USA

**Keywords:** tobacco, *NIC1*, ERF, gene editing, low nicotine

Nicotine is the predominant alkaloid in tobacco plants, accounting for ~90% of their total alkaloid content. It is the main addictive substance in cigarettes. Reducing nicotine content in tobacco leaves will aid the development of low‐nicotine tobacco products. Prior work has shown that the manipulation of genes involved in nicotine biosynthesis can achieve this purpose (Hidalgo Martinez *et al*., 2020). Here, we focussed on the long‐sought major regulator of nicotine biosynthesis, *NIC1* (*A*).

The *NIC1* gene, together with a minor locus *NIC2* (*B*), have been identified through genetic analysis of a low‐nicotine trait originating from natural mutants of cigar tobacco (Legg and Collins, 1971). Introgression of the low‐nicotine trait into Burley 21 (B21) generated near‐isogenic lines with different alkaloid levels: high alkaloid (HA, *AABB*), high intermediate (HI, *AAbb*), low intermediate (LI, *aaBB*) and low alkaloid (LA, *aabb*) (Legg and Collins, 1971). The genes coding for nicotine biosynthetic enzymes, such as the rate‐limiting PMT and QPT, are downregulated in LA, suggesting that *NIC* genes are transcriptional regulators orchestrating nicotine biosynthesis (Saunders and Bush, 1979). Transcriptome‐based cloning of *NIC2* revealed that this locus is clustered with transcription factors from the ethylene response factor (ERF) subfamily. Of these *NIC2‐ERFs*, *ERF189* is the most effective and directly targets the GC‐rich P‐box element in promoters of nicotine biosynthetic genes (Shoji and Hashimoto, 2012; Shoji *et al*., 2010). Suppression of *NIC2*‐*ERF*s reduced nicotine content in tobacco, but a significant amount of nicotine remained due to the major *NIC1* locus (Kajikawa *et al*., 2017).

To isolate the *NIC1* gene, we conducted map‐based cloning using 600 field‐growing F_2_s derived from a cross between HI and LA. The segregating population was first genotyped with a custom tobacco 30K Infinium iSelect HD BeadChip. Additional markers were designed through SNP identification based on RNA‐seq of the B21 NILs. *NIC1* congregated with SNP4 and was flanked by SNP3 and SNP5 on chromosome 7 (Figure 1a). The delimited *NIC1* region was bordered by K326 scaffolds Nitab4.5_0003553 and Nitab4.5_0007027. Reciprocal BLAST comparisons were conducted between K326 and TN90 to fill gaps. Gene annotation identified at least seven full‐length single‐exon ERFs (*JRE5L2*, *ERF199*, *ERF91*, *ERF210*, *ERF29*, *ERF16* and *ERF130*) and two truncated ERFs (*ERF110* and *ERF17L3ΔN*) in this region (Figure 1a).

**Figure 1:**
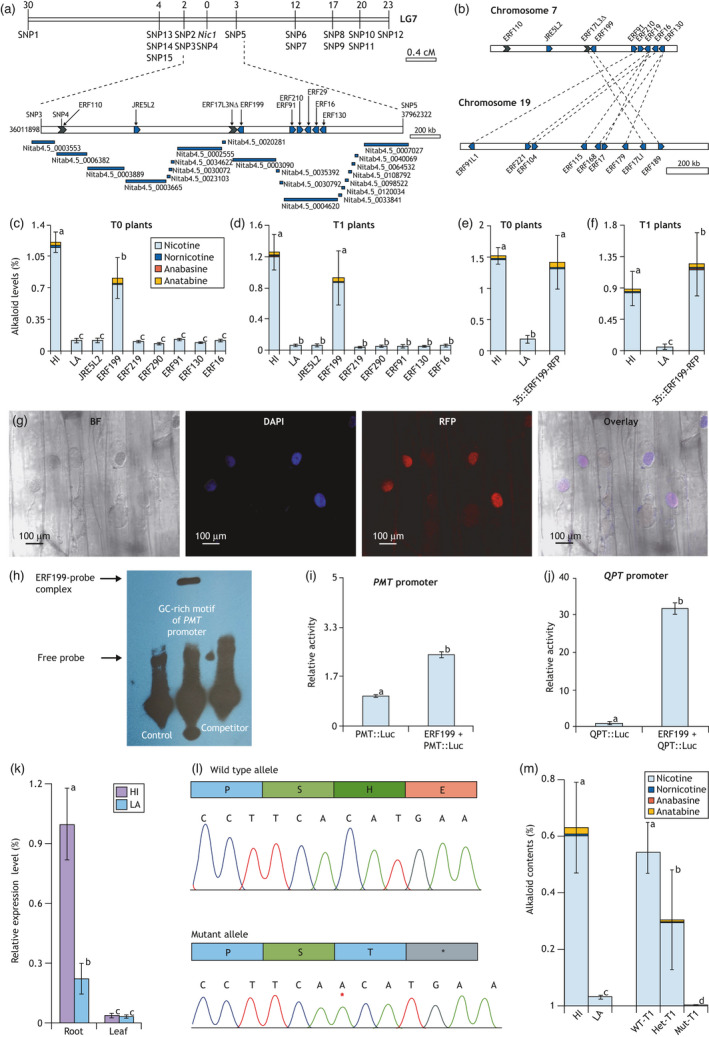
Map‐based cloning and functional characterization of *NIC1*. (a) Genetic mapping localizes *NIC1* onto LG7. Numbers above linkage group indicate recombination events. ERF‐encoding genes shown on chromosome. Arrows represent the transcriptional directions. Grey arrows represent incomplete *ERFs*. (b) The *NIC1* region on Chr. 7 is homologous to *NIC2* on Chr. 19. (c–f) Alkaloid levels in transgenic LA plants at T0 and T1. *ERF199* driven by either the native (c, d) or 35S promoters (e, f) significantly increases alkaloid levels. (g) Root cells overexpressing ERF199‐RFP visualized with Olympus FV1000 confocal microscope. Localization of ERF199 to the nucleus apparent in overlay of bright field (BF), DAPI, and RFP images. Scale bars: 100 µm. (h) EMSA and competition experiment indicated the direct binding of ERF199 to the P‐box element in the *PMT2* promoter. Competitor is 1000× concentrated probe without biotin labelling. (i, j) ERF199 causes significant induction of Luciferase reporter driven by the *PMT2* (i) or *QPT* (j) promoters in tobacco BY‐2 protoplasts. (k) qRT‐PCR reveals downregulation in LA and root specificity of *ERF199*. (l) Introduction of an ‘A’‐insertion (red asterisk) into *NIC1* in HI by gene editing causes a premature stop codon. (m) Loss of ERF199 function dramatically reduces alkaloid levels in HI at T1. Different letters on bar graphs indicate significant at 0.01 level by Tukey’s test.

BLAST analysis showed that *NIC1* and *NIC2* regions were syntenic and originated, respectively, from *N. sylvestris* (S‐genome) and *N. tomentosiformis* (T‐genome). Notably, ERF199 (Nitab4.5_0003090g0030) is homologous to ERF189, sharing an identical binding domain (Figure 1b). Driven by their native promoters, the seven complete *NIC1‐ERFs* were transferred to LA plants using *Agrobacterium tumefaciens* strain GV3101. Complementation tests showed that transfer of *ERF199* (*n* = 28) significantly increased nicotine levels in potted T0 plants growing in greenhouse. No significant phenotype changes resulted from the remaining six *NIC1‐ERFs* (*n* > 20) (Figure 1c). The *ERF199*‐mediated nicotine increase was confirmed with T1 plants (*n* = 35 from 7 T0s) (Figure 1d). We, therefore, concluded that *ERF199* is the *NIC1* gene.

To determine subcellular localization, we fused ERF199 with a red fluorescent protein (RFP) under the CaMV 35S promoter. Constitutive expression of ERF199‐RFP in LA significantly increased nicotine content in both T0 (*n* = 15) and T1 (*n* = 28) plants (Figure 1e, f). Co‐localization of the fusion protein with 4′, 6‐diamidino‐2‐phenylindole (DAPI)‐stained nuclei demonstrated that ERF199 is localized in the nucleus, consistent with its role as a transcriptional regulator (Figure 1g). Direct binding of ERF199 to the P box in the *PMT2* promoter was verified by electrophoretic mobility shift assay (EMSA). The resulted mobility shift was eliminated by the addition of excess non‐labelled probe in the competition experiment, confirming the specificity of this DNA–protein interaction (Figure 1h). Furthermore, a transient gene expression assay using tobacco protoplasts revealed that ERF199 regulates both PMT2 and QPT, the two key enzymes for nicotine biosynthesis (Figure 1i, j).

We compared the genomic sequences of *EFR199* alleles between HI and LA (~5kb), including 2.3kb upstream of the start codon and 2kb downstream of the stop codon, but no SNPs were detected. Expression analysis indicated that *Nic1* was root‐specific and significantly downregulated in LA (Figure 1k), suggesting the recessive allele was epigenetically silenced. However, targeted bisulphite sequencing of the same 5kb did not reveal significantly different DNA methylation in CpG, CHG and CHH sites. A thorough epigenome sequencing may provide insight into the underlying epimutations in the *NIC1* locus.

We predicted that lower nicotine than LA could be attained by eliminating *ERF199*. Using CRISPR technology, we generated a mutated allele caused by an ‘A’ insertion in HI (Figure 1l) and evaluated its phenotypic effect at T1. The genotypes of wild‐type (WT‐T1), heterozygous (Het‐T1) and homozygous mutant (Mut‐T1) plants were determined by DNA sequencing. Total alkaloid levels in WT‐T1 plants (*n* = 22) were comparable with HI plants, and Het‐T1s (*n* = 29) had intermediate levels. However, alkaloid content was barely discernible in Mut‐T1 plants (*n* = 35); approximately 1/10 of that in LA plants (Figure 1m). Thus, manipulation of the *NIC1* gene provides a new strategy for nicotine control. Furthermore, the ultra‐low‐nicotine levels in Mut‐T1 confirmed that *ERF199* is the only causal gene for nicotine biosynthesis within the *NIC1* locus.

Transcriptional regulation of secondary metabolite production can be controlled by a single *ERF*. Indeed, *GAME9* locates within an *ERF* cluster and is the only functional regulator of steroidal glycoalkaloid biosynthesis in tomatoes (Cardenas *et al*., 2016). Although both ERF189 and ERF199 share the same DNA‐binding domain and directly bind to P‐box elements within the promoters, ERF199 is more effective. A plausible explanation may be the presence of cofactor/coactivator‐recruiting activation domains. Further investigation is required to determine whether a unique activation domain or additional transcriptional cofactors play critical roles in the ERF199‐regulatory network.

Significant endeavours have been made to attenuate tobacco nicotine content (Hidalgo Martinez *et al*., 2020). Our study genetically and functionally validated *ERF199* as the *NIC1* gene. The *NIC1* locus, originating from the S‐genome, is homologous to the T‐genome‐donated *NIC2* locus. Constitutive expression of *ERF199* caused increased nicotine levels, and disruption of ERF199 function in the absence of *NIC2* dramatically reduced nicotine accumulation in leaves. Thus, genetic regulation of nicotine levels in tobacco plants can be achieved by manipulating the *NIC1* gene.

## Conflicts of interest

The authors declare that there are no conflicts of interest.

## Author contributions

MH, TG, AF, DL and SY conceived and designed the experiments. All authors performed the experiments. SY wrote the first draft of the manuscript. All authors revised the manuscript.

## Accession numbers

Sequences in this article can be found in GenBank under accession numbers MZ541068 and MZ768806‐MZ768811.

## References

[pbi13694-bib-0001] Cardenas, P.D. , Sonawane, P.D. , Pollier, J. , Vanden Bossche, R. , Dewangan, V. , Weithorn, E. , Tal, L. *et al*. (2016) GAME9 regulates the biosynthesis of steroidal alkaloids and upstream isoprenoids in the plant mevalonate pathway. Nat. Commun. 7, 10654. 26876023 10.1038/ncomms10654 PMC4756317

[pbi13694-bib-0002] Hidalgo Martinez, D. , Payyavula, R.S. , Kudithipudi, C. , Shen, Y. , Xu, D. , Warek, U. , Strickland, J.A. *et al*. (2020) Genetic attenuation of alkaloids and nicotine content in tobacco (*Nicotiana tabacum*). Planta, 251, 92. 32242247 10.1007/s00425-020-03387-1

[pbi13694-bib-0003] Kajikawa, M. , Sierro, N. , Kawaguchi, H. , Bakaher, N. , Ivanov, N.V. , Hashimoto, T. and Shoji, T. (2017) Genomic insights into the evolution of the nicotine biosynthesis pathway in tobacco. Plant Physiol. 174, 999–1011. 28584068 10.1104/pp.17.00070 PMC5462024

[pbi13694-bib-0004] Legg, P.D. and Collins, G.B. (1971) Inheritance of percent total alkaloids in *Nicotiana tabacum* L. II. Genetic effects of 2 loci in Burley 21 x La Burley 21 populations. Can. J. Genet. Cytol. 13, 287–291.

[pbi13694-bib-0005] Saunders, J.W. and Bush, L.P. (1979) Nicotine biosynthetic enzyme activities in *Nicotiana tabacum* L. Genotypes with different alkaloid levels. Plant Physiol. 64, 236–240. 16660940 10.1104/pp.64.2.236 PMC543062

[pbi13694-bib-0006] Shoji, T. and Hashimoto, T. (2012) DNA‐binding and transcriptional activation properties of tobacco NIC2‐locus ERF189 and related transcription factors. Plant Biotechnol. 29, 35–42.

[pbi13694-bib-0007] Shoji, T. , Kajikawa, M. and Hashimoto, T. (2010) Clustered transcription factor genes regulate nicotine biosynthesis in tobacco. Plant Cell, 22, 3390–3409. 20959558 10.1105/tpc.110.078543 PMC2990138

